# O.2.1-8 Who are suitable targets for a social prescribing intervention addressing physical activity? A survey-based study including viewpoints from general practitioners

**DOI:** 10.1093/eurpub/ckad133.113

**Published:** 2023-09-11

**Authors:** Mette Rod Christensen, Halfdan Thorsø Skjerning, Knud Ryom, Julie Sandell Jacobsen, Lene Gissel Rasmussen, Lasse Greve Hansen, Rasmus Østergaard Nielsen

**Affiliations:** Research Unit for General Practice, Aarhus, Denmark; Research Unit for General Practice, Aarhus, Denmark; Department of Public Health, Aarhus University, Denmark; Research Unit for General Practice, Aarhus, Denmark; Research Centre for Health and Welfare Technology, VIA University College, Aarhus; metterodchristensen@hotmail.com; Research Unit for General Practice, Aarhus, Denmark; Department of Public Health, Aarhus University, Denmark; Research Unit for General Practice, Aarhus, Denmark; Research Unit for General Practice, Aarhus, Denmark; Department of Public Health, Aarhus University, Denmark

## Abstract

**Purpose:**

Social prescribing has been suggested as an inter-sectoral intervention to increase physical activity. In the intervention, healthcare professionals refer patients or citizens to a link worker who is assisting them to locate peer-based activities in their local community. The present study investigated which groups of physically inactive adults the Danish general practitioners reported as relevant target groups for a social prescribing intervention to increase the level of physical activity.

**Methods:**

The study design was a descriptive survey-based study. Danish general practitioners completed an online survey. On 10-point interval scales, they rated the suitability of referring 25 selected target groups of physically inactive adults (18+) with medical conditions or health-threatening behaviour to a social prescribing intervention to increase their level of physical activity. A score of 0 referred to not suitable at all, whereas a score of 10 indicated highly suitable. Using non-parametric statistics, target groups were considered eligible for an intervention if the 25th percentile had a score of 8 or above indicating that 75% of the GPs reported a score of 8 or above.

**Results:**

A total of 52 general practitioners completed the survey. Of these, 75% reported a score of 8 or above to Danes with metabolic syndrome (median=10; 25th percentile=9), overweight (median=10; 25th percentile=9), diabetes (median=10; 25th percentile=8), and low back pain (median=9; 25th percentile=8) indicating these to be target groups of social prescribing to increase the level of physical activity. GPs considered the following groups less suitable for an intervention: persons with eating disorders (median=4; 25th percentile=2), migraine (median=5; 25th percentile=3), and women during postpartum (median=6; 25th percentile=3). Data from open-ended questions added nuances to the ratings and suggested citizens struggling with loneliness to be a new target group for social prescribing.

**Conclusions:**

A majority of the responding Danish GPs identified persons with metabolic syndrome, overweight, diabetes, and low back pain as being target groups suited for a social prescribing intervention to increase the level of physical activity. These results are interesting since several approaches to impede the level of physical inactivity exist, and political and organizational efforts have become subject of the recent attention.

